# Assessment of Elastic Fibers in Tumor Stroma as a New Method to Predict 6-Year Outcomes for Gastric Cancer Patients

**DOI:** 10.3389/fonc.2020.00395

**Published:** 2020-04-21

**Authors:** Xiaowei Xue, Yuan Li, Yuhan Zhang, Pengyan Wang, Xiaoqing Li, Hongtao Wei, Weixun Zhou

**Affiliations:** ^1^Department of Pathology, Molecular Pathology Research Center, Peking Union Medical College Hospital, Chinese Academy of Medical Science, Beijing, China; ^2^Department of Gastroenterology, Peking Union Medical College Hospital, Chinese Academy of Medical Science, Beijing, China; ^3^Department of Gastroenterology, Beijing Friendship Hospital, Capital Medical University, Beijing, China

**Keywords:** elastic fibers, tumor stoma, histochemistry, gastric cancer, prognosis

## Abstract

**Purpose:** This study aimed to determine whether detecting elastic fibers in tumor stroma (EFTS) could be used as a new method for predicting the prognosis of gastric cancer (GC) patients.

**Materials and Methods:** EFTS expression was determined by histochemistry in 160 GC patients who underwent gastrectomy. Based on the staining results, the patients were divided into three groups according to their EFTS expression level: low (*n* = 57), moderate (*n* = 50), and high (*n* = 53). The clinicopathological data and 6-year survival data were analyzed among different EFTS groups.

**Results:** The expression of EFTS was closely related to lymphovascular invasion (*P* = 0.010), blood transfusion in operation (*P* < 0.001), recurrence rate (*P* < 0.001), and motility (*P* < 0.001). High expression of EFTS was also correlated with recurrence-free survival (RFS) and overall survival (OS) in GC patients by Kaplan–Meier curve (*P* < 0.001 for RFS and *P* < 0.001 for OS).

**Conclusions:** Multivariate analysis showed that EFTS was an independent prognostic factor for RFS and OS. In conclusion, detecting EFTS to predict the prognosis of GC patients is effective and highly feasible.

## Introduction

Gastric cancer (GC) is recognized as the fifth most common cancer and the third leading cause of cancer-related deaths worldwide (1). A point worth emphasizing is that half of GCs all over the world occur in countries located in Eastern Asia (2, 3), such as China and Japan; therefore, it is more urgent to research the diagnosis and treatment of GC in these areas compared to others. With the development of endoscopic technology, the early detection of GC has become possible. Unfortunately, a large number of patients are still diagnosed with GC at an advanced stage. For advanced GC patients, although neoadjuvant therapy and postsurgery treatment, such as chemo and/or radiation therapy, are widely used, radical gastrectomy is still the first choice for patients who qualify; however, the postoperative prognosis of each patient varies greatly (4). Until now, there has been no good prognostic marker for GC except for TNM staging. Therefore, it is of great clinical significance to identify new prognostic biomarkers or methods for GC patients.

Produced by fibroblasts and smooth muscle cells, elastic fiber (EF) is one of the extracellular interstitial components in connective tissue, and its main function is to maintain tissue elasticity (5). Currently, EF has been evaluated in many malignant tumors, including lung cancer (6), colorectal cancer (7), and melanoma (8), to determine whether there is vascular invasion, assess the depth of tumor invasion, and differentiate certain types of tumors (6, 7, 9, 10). However, few studies investigate EFs in the peritumoral tissues of GC. In our practice, we have observed that the amount of EFs in peritumoral tissues varies between different GC cases, but its clinical significance is still unclear.

The aim of this study was to determine whether detecting EFs in tumor stroma (EFTS) could be used as a new marker to predict the outcomes of GC patients who underwent radical gastrectomy.

## Materials and Methods

### Patients and Tissue Samples

This study involved a retrospective review of the pathological records, specimens, and follow-up information from 160 patients in the Department of Pathology at Peking Union Medical College Hospital between January 2013 and December 2015. These patients underwent gastrectomy, were diagnosed with gastric adenocarcinoma, and had enough resected lymph nodes (≥15). Exclusion criteria included (1) recurrent stomach cancer; (2) chronic infections or history of inflammatory disease, including vasculitis, systemic lupus erythematosus (SLE), and rheumatologic condition; (3) neoadjuvant therapy or any cancer-related surgery; (4) cancer arising from the gastroesophageal junction; and (5) other synchronous malignancies.

Clinical data included patient age at the time of surgery, gender, tumor site and size, surgical procedures, lymph nodes status, and follow-up data. Two experienced gastrointestinal (GI) pathologists (LY and ZW) reviewed each case independently. The clinical pathological features included pathologic type, differentiation degree, perineural invasion, lymphovascular invasion, and lymph node metastasis. If there was discordance, a third GI pathologist was consulted. Tumor stages of I–IV were assigned according to the TNM staging system of GC (American Joint Committee on Cancer, Eighth Edition). The invasion patterns were divided according to the Japanese classification system (11). For each specimen, we took 3–7 pieces of tumor tissue according to the size of the tumor. Differentiation was performed by dividing cases into three groups: well-differentiated, moderately differentiated, and poorly differentiated adenocarcinoma (12).

### Follow-Up of Patients

Enrolled patients underwent computed tomography or gastroscope review in real-life clinic practice by outpatient clinic or telephone at least every 6 months. Follow-up data were collected from the time of surgery to 6 years after operation and were presented as recurrence-free survival (RFS) and overall survival (OS). RFS was defined as the time between the operation and tumor recurrence or death caused by GC. Tumor recurrence was considered local recurrence or distal metastasis in pathological or imaging findings. OS was defined as the time from operation until death by any cause. In total, 29 patients (18.13%) were lost to follow-up. Seventy patients (43.76%) experienced recurrence, and 74 (46.25%) patients died during 6 years of follow-up.

### Elastic Staining

Using a rotary microtome, labeled and adhesive sections were cut at 4 μm. All sections were dewaxed with xylene, hydrated in descending concentrations of alcohol (100, 90, and 70%), followed by distilled water for 2 min each. Sections were oxidized by KmnO_4_ and reduced by oxalic acid, washed twice with distilled water, then incubated with Victoria blue solution for 2 h. Samples were then rinsed in 95% alcohol to remove iodine staining, counterstained in van Gieson stain for 30 s, dehydrated in absolute alcohol, cleared in xylene, and mounted with synthetic resin (13). The EFs were stained blue–green, collagen was stained red, and other tissue elements were stained yellow.

### Data Recording

All EF slides were examined under the light microscope by two histopathologists using 10 and 40× magnifications, and the results were recorded. The amount of EFTS (EFs in the peripheral area of the tumor) was evaluated and classified as (10): (1) low grade: no EFs were displayed in tumor stroma, or fine fibrils were distributed randomly around blood vessels; (2) moderate grade: there were EFs demonstrable in tumor stroma, but EFs were present in a small amount and were not connected with each other; (3) high grade: there was a large amount of EFs displayed in tumor stroma, and EFs were connected to each other.

### Statistical Analysis

Based on the elastic staining results, patients from the three groups were compared in terms of demographics and the pathological characteristics of tumors. To analyze categorical variables, the χ^2^ test was used. For survival analysis, the Kaplan–Meier method was adopted, and to analyze the differences in survival, the log-rank test was used. The Cox proportional hazards model was built for multivariate analysis. To perform statistical analyses, SPSS 25.0 (SPSS for Windows, SPSS Inc., Chicago, IL) was adopted. A *P* < 0.05 indicated significant differences.

## Results

### Patient Clinicopathologic Features

All clinicopathologic data are detailed in Supplementary Table 1. Among the 160 GC patients enrolled in this study, there were 104 (65.00%) males and 56 (35.00%) females. The age of the patients ranged from 31 to 88 years old, the median age was 61 years old, and 78 (48.75%) patients were under 60 years old. The tumor size was <3 cm in 46 (28.75%) patients, 3–5 cm in 72 (45.00%) patients, and was >5 cm in 42 (26.25%) patients. Fifteen (9.38%) patients had a family history of cancer, and 82 (51.25%) patients reported weight loss at the time of GC diagnosis. Further, 7 (4.38%) tumors were located at the cardia, 35 (21.88%) were at the body/fundus, 109 (68.13%) were at the antrum, and 9 (5.63%) involved the whole stomach. In addition, 20 (12.50%) GC cases were well-differentiated, 51 (31.88%) were moderately differentiated, and 89 (55.63%) cases were poorly differentiated. Lymphovascular invasions were found in 14 (8.75%) patients. Intestinal types were identified in 66 (41.25%) patients according to Lauren Classification, and INFc types were found in 113 (70.63%) patients according to the invasive pattern. According to Klintrup–Makinen (K-M) grade and tumor stroma percentage (TSP), the number of people in the low group is 136 (85.00%) and 145 (90.63%), respectively. Fifty-seven (35.63%) patients were given a blood transfusion during the surgery.

### Histochemical Staining of Elastic Fibers in Tumor Stroma in Gastric Cancer Patients

**Figure 1:**
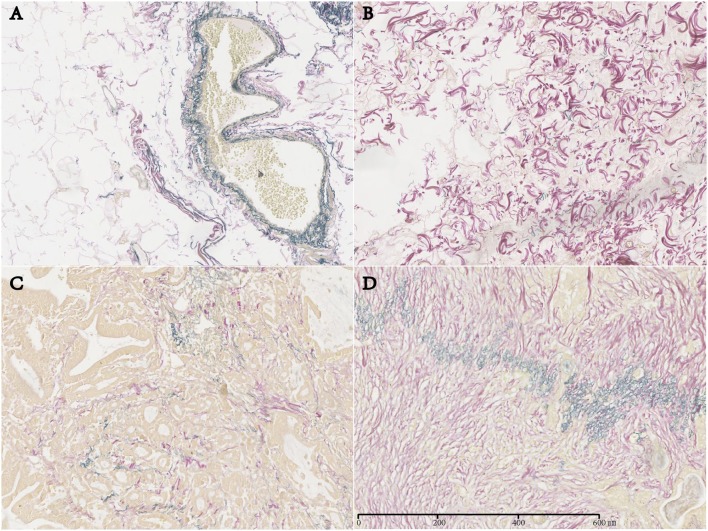
Expression of elastic fibers (EFs) in normal gastric tissue and EFs in tumor stroma (EFTS) in different gastric cancer tissues (magnification, ×400). **(A)** EF in normal gastric tissue. **(B)** EFTS low expression. **(C)** EFTS moderate expression. **(D)** EFTS high expression.

In normal stomach tissue samples, EFs were regularly distributed and were mainly located in the mucosal muscle layer and the muscle layer adjacent to the submucosal side (Figure 1A). However, in GC tissue, EFTS was characteristically distributed mainly near the perivascular area, smooth muscle bundle, and peripheral area of the cancer nest (Figures 1B–D). Among 160 GC patients, there were 53 cases (33.13%) with high EFTS expression, 50 cases (31.25%) with moderate EFTS expression, and 57 cases (35.63%) with low EFTS expression.

### Correlation Between Elastic Fibers in Tumor Stroma Expression and Clinicopathologic Features

Clinicopathologic data of all patients were compared between the three EFTS expression groups according to the histochemistry results, as demonstrated in Supplementary Table 1. No significant difference was found among the three groups in terms of sex, age, tumor size, primary lesion location, surgical mode, Lauren pathological type, invasion pattern, K-M grade, TSP, or differentiation degree; however, lymphovascular invasion (*P* = 0.010), blood transfusion during operation (*P* < 0.001), recurrence rate (*P* < 0.001), and motility (*P* < 0.001) were significantly different between the three groups.

### Relationship Between Elastic Fibers in Tumor Stroma and Prognosis

**Figure 2:**
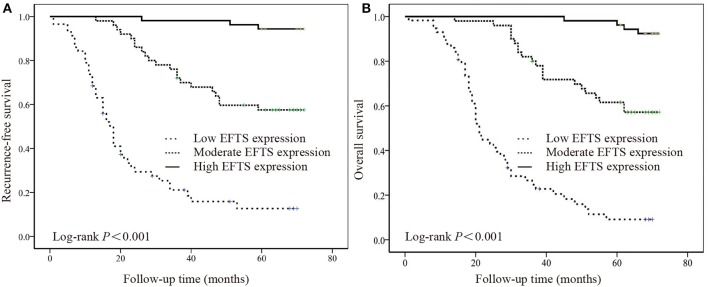
The relationship between the expression of elastic fibers in tumor stroma (EFTS) and survival. Kaplan–Meier curve for **(A)** recurrence-free survival (RFS) and **(B)** overall survival (OS) for different EFTS expressions.

The relationships between RFS and OS values and EF expression are summarized in Supplementary Table 2. Five parameters, including sex (*P* = 0.040 and *P* = 0.049), loss of weight (*P* = 0.001 and *P* < 0.001), lymphovascular invasion (*P* < 0.001 and *P* = 0.001), WHO performance status (*P* = 0.044 and *P* = 0.031), and the expression of EFTS (*P* < 0.001 and *P* < 0.001), were significantly related to RFS and OS, respectively. Additionally, lymphovascular invasion (*P* = 0.042) and EFTS expression (*P* < 0.001) were independent prognostic factors for RFS, and EFTS expression (*P* < 0.001) was an independent prognostic factor for OS. The Kaplan–Meier curve and log-rank test also indicated that EFTS expression was associated with RFS and OS in GC (*P* < 0.001; Figures 2A,B).

## Discussion

Elastin staining is widely used in determining tumor serosal invasion. It has been recognized that patients with serosal invasion have poor prognosis in many types of carcinoma, including lung, gastric, and colorectal cancers (7, 9, 14). Recently, the roles of the tumor stroma and stromal reaction in modulating the growth and invasion of the tumor have received more attention (10, 15). The phenomenon of hyperplastic degeneration of EFTS was first noted in breast cancer, and later in parotid tumors. In breast cancer, regardless of whether it was invasive ductal carcinoma or lobular carcinoma, studies showed that patient prognosis with EFTS expression was better than in those without EFTS expression (16). Consistent with their findings, we found that EFTS is associated with prognosis in GC, thus confirming the important role of EFTS in tumor disease.

These findings suggest that detection of EFTS could be a potential predictor of prognosis in GC patients after radical treatment, and that patients in the EFTS high expression group have a better prognosis than those in the low expression group. Our conclusions are derived from three aspects. First, we found that EFTS expression is inversely associated with vascular infiltration in patients with GC, and vascular infiltration is a pathological hallmark of tumor recurrence and metastasis. Second, patients with a high EFTS expression had lower recurrence and mortality rates after radical resection. Survival curves showed significant differences between EFTS expression subgroups. Finally, univariate and multivariate analyses found that EFTS was an independent risk factor for RFS and OS. Based on our findings, we recommend that patients with a low EFTS expression be closely monitored or treated prophylactically to improve long-term outcomes in patients with GC.

Our research is different from previous studies, which mainly focused on the relationship between the integrity of organic serous EF membrane and the distant metastasis of tumors. Conversely, this study is mainly concerned with the relationship between scattered EF (EF among cancer nests) and the prognosis of tumors. Notably, we did not see EF in various types of GC tissues, indicating that cancer tissues themselves could not synthesize EF. Therefore, we speculate that the possible mechanism of EFTS production in paracancerous tissues involves the cancer cells releasing select cytokines, promoting the generation of EFTS in smooth muscle cells, fibroblasts, and other cells adjacent to the tumor tissue in a paracrine manner. We speculate that the EFTS response is a defense mechanism against tumors in the carcinoma stroma and that EFTS may act to isolate tumors from normal tissues and protect against tumor metastasis and invasion. A low expression of EFTS indicates that the protective barrier against tumor progression is deficient, allowing the tumor to progress easier and resulting in a poorer prognosis.

To better illustrate our results, two stroma parameters were included in the study, TSP and K-M grade. TSP represents the relative amount of tumor stroma, and K-M grade correlates with the density of inflammatory cells. These two parameters can reflect the overall situation of tumor stroma. In addition, they were reported to provide prognostic values in colorectal cancers (17, 18). Our results suggested that these two parameters had neither correlation with EFTS expression nor correlation with RFS and OS in these GC cases.

Invasive patterns, like Japanese INF classification, have been reported to be related to prognoses of GC patients. Patients with a higher INF classification usually have worse prognosis. Our results showed no correlation between infiltrative pattern. That may be because we had only 113 patients (70.63%) in our cohort. If we expand the sample size, the INF classification may show a prognostic value. Our results showed that INF data had no correlation with EFTS expression either. These results suggest that the prognostic value of EFTS may have a relation to the body's self-protection on tumor growth, rather than the tumor's invasive ability.

Interestingly, EFTS was negatively correlated with blood transfusion. We believe that this result may be related to EFTS and tumor infiltration, vascular infiltration, and tumor blood supply; however, the exact causal relationship needs further investigation.

So many genes/protein markers have been explored that can make a judgment on the prognosis of GC. But they need expensive equipment, super technology, and long-term testing. EFTS is easy in daily work. And sometimes staining for EFTS is not an extra work for EF staining can help to judge venous invasion at the same time. In addition, the mechanisms on why and how protective effects of EFTS on the progression of GC worked are unclear, which can also provide new directions for future basic scientific research.

A small sample size was the main drawback of this study. A more comprehensive analysis is needed to determine more risk factors for determining poor OS and RFS, such as adjuvant therapy, molecular markers, and cell signaling transduction. It also needs to be further evaluated in another GC validation cohort.

## Conclusion

In conclusion, our findings indicate that detecting EFTS may be regarded as a novel method for predicting the outcomes of GC patients after radical resection. It is also suggested that after radical resection, patients with lower EFTS should be closely monitored and given prophylactic treatment.

## Data Availability Statement

The datasets generated for this study are available on request to the corresponding author.

## Ethics Statement

The studies involving human participants were reviewed and approved by Peking Union Medical College Hospital Ethics Review Committee. Written informed consent for participation was not required for this study in accordance with the national legislation and the institutional requirements. Written informed consent was not obtained from the individuals for the publication of any potentially identifiable images or data included in this article.

## Author Contributions

WZ and HW designed the study and contributed to its conception. XX and HW were major contributors in the writing of the manuscript. XX, YL, and YZ performed the previous experiments. PW and XL checked the experimental data and provided advice. All authors read and approved the final manuscript.

## Conflict of Interest

The authors declare that the research was conducted in the absence of any commercial or financial relationships that could be construed as a potential conflict of interest.

## Abbreviations

EFTS: elastic fibers in tumor stroma
GC: gastric cancer
OS: overall survival
RFS: recurrence-free survival
HC: histochemistry
EF: elastic fiber.

## References

[1] Uyama I Suda K Nakauchi M Kinoshita T Noshiro H Takiguchi S. Clinical advantages of robotic gastrectomy for clinical stage I/II gastric cancer: a multi-institutional prospective single-arm study. Gastric Cancer. (2018) 22:377–85. 10.1007/s10120-018-00906-8 30506394

[2] Ferlay J Soerjomataram I Dikshit R Eser S Mathers C Rebelo M. Cancer incidence and mortality worldwide: sources, methods and major patterns in GLOBOCAN 2012. Int J Cancer. (2015) 136:E359–86. 10.1002/ijc.29210 25220842

[3] Ajani JA D'Amico TA Almhanna K Bentrem DJ Chao J Das P. Gastric cancer, version 3.2016, NCCN clinical practice guidelines in oncology. J Natl Compr Cancer Network. (2016) 14:1286–312. 10.6004/jnccn.2016.0137 27697982

[4] Van Cutsem E Sagaert X Topal B Haustermans K Prenen HJTL. Gastric cancer. Lancet. (2016) 388:2654–64. 10.1016/S0140-6736(16)30354-3 27156933

[5] Wagenseil JE Mecham RP. New insights into elastic fiber assembly. Birth Defects Res C Embryo Today. (2007) 81:229–40. 10.1002/bdrc.20111 18228265

[6] Huang H Wang T Hu B Pan C. Visceral pleural invasion remains a size-independent prognostic factor in stage I non-small cell lung cancer. Ann Thorac Surg. (2015) 99:1130–9. 10.1016/j.athoracsur.2014.11.052 25704861

[7] Lu J Hu X Meng Y Zhao H Cao Q Jin M. The prognosis significance and application value of peritoneal elastic lamina invasion in colon cancer. PLoS ONE. (2018) 13:e0194804. 10.1371/journal.pone.0194804 29630617 PMC5890982

[8] Stillhard A Cazzaniga S Borradori L Beltraminelli H. Pushing and loss of elastic fibers are highly specific for melanoma and rare in melanocytic nevi. Arch Dermatol Res. (2019) 311:99–107. 10.1007/s00403-018-1885-x 30547366

[9] Lei G Yang H Hong T Zhang X Yang N Zhang Y Elastic staining on paraffin-embedded slides of pT3N0M0 gastric cancer tissue. J Vis Exp. (2019) 147:e58278 10.3791/58278 31107435

[10] Zakout YM Abdullah SM Ali MA. Assessment of elastosis in invasive ductal carcinoma of the breast compared to fibroadenoma among Sudanese patients using conventional histochemical methods. Biotech Histochem. (2012) 87:122–5. 10.3109/10520295.2011.565805 21375429

[11] Japanese Gastric Cancer Association Japanese classification of gastric carcinoma: 3rd English edition. Gastric Cancer. (2011) 14:101–12. 10.1007/s10120-011-0041-5 21573743

[12] WHO Classification of Tumours Editorial Board Digestive System Tumours, Classification of Tumours. 5th ed Vol 1 (2019).

[13] Guan L Xu G. Damage effect of high-intensity focused ultrasound on breast cancer tissues and their vascularities. World J Surg Oncol. (2016) 14:153. 10.1186/s12957-016-0908-3 27230124 PMC4882851

[14] Unnikandam Veettil SR Van Bruggen SM Hwang DG Bartlett MD Schneider IC. Tuning surface functionalization and collagen gel thickness to regulate cancer cell migration. Colloids Surf B Biointerfaces. (2019) 179:37–47. 10.1016/j.colsurfb.2019.03.031 30933893

[15] Fu H Yang H Zhang X Xu W. The emerging roles of exosomes in tumor-stroma interaction. J Cancer Res Clin Oncol. (2016) 142:1897–907. 10.1007/s00432-016-2145-0 26987524 PMC11819208

[16] Rasmussen BB Pedersen BV Thorpe SM Rose C. Elastosis in relation to prognosis in primary breast carcinoma. Cancer Res. (1985) 45:1428–30. 3971386

[17] Park JH McMillan DC Powell AG Richards CH Horgan PG Edwards J. Evaluation of a tumor microenvironment-based prognostic score in primary operable colorectal cancer. Clin Cancer Res. (2015) 21:882–8. 10.1158/1078-0432.CCR-14-1686 25473000

[18] Park JH van Wyk H Roxburgh CSD Horgan PG Edwards J McMillan DC. Tumour invasiveness,the local and systemic environment and the basis of staging systems in colorectal cancer. Br J Cancer. (2017) 116:1444–50. 10.1038/bjc.2017.108 28427085 PMC5520088

