# Supplementary material for: Assessment of Elastic Fibers in Tumor Stroma as a New Method to Predict 6-Year Outcomes for Gastric Cancer Patients
Source: Front Oncol. 2020 Apr 21;10:395. doi: 10.3389/fonc.2020.00395 (PMC7186317; doi:10.3389/fonc.2020.00395)
Supplement: Supplementary file 1 [file Table_1.DOCX]

Table 1. Correlation between elastic fibers in tumor stroma (EFTS) expression and clinicopathologic variables of patients with gastric cancer

| Variables | Total (n=160) | Expression of elastic fibers | | | P-value |
| --- | --- | --- | --- | --- | --- |
|  |  | Low(n=57) | Moderate(n=50) | High(n=53) |  |
| Sex  Male  female | 104 (65.00)  56 (35.00) | 43 (75.44)  14 (24.56) | 29 (58.00)  21 (42.00) | 32 (60.38)  21 (39.62) | 0.116 |
| Age  ≤60  ＞60 | 78 (48.75)  82 (51.25) | 27 (47.37)  30 (52.63) | 25 (50.00)  25 (50.00) | 26 (49.06)  27 (50.94) | 0.962 |
| Size  ＜3  3-5  ＞5 | 46 (28.75)  72 (45.00)  42 (26.25) | 17 (29.82)  26 (45.61)  14 (24.56) | 17 (34.00)  22 (44.00)  11 (22.00) | 12 (22.64)  24 (45.28)  17 (32.08) | 0.313 |
| Family  negative  positive | 145 (90.63)  15 (9.38) | 51 (89.47)  6 (10.53) | 44(88.00%)  6 (12.00) | 50 (94.34)  3 (5.66) | 0.508 |
| Lose weight  negative  positive | 78 (48.75)  82 (51.25) | 22 (38.60)  35 (61.4) | 24 (48.00)  26 (52.00) | 32 (60.38)  21 (39.62) | 0.073 |
| location  cardia  gastric body  [antrum](javascript:void(0);)  total stomach | 7 (4.38)  35 (21.88)  109 (68.13)  9 (5.63) | 3 (5.26)  16 (28.07)  33 (57.89)  5 (8.77) | 3 (6.00)  11 (22.00)  34 (68.00)  2 (4.00) | 1 (1.89)  8 (15.09)  42 (79.25)  2 (3.77) | 0.353 |
| hospital days | 19.00 (16.00, 25.00) | 20.00 (17.00, 26.00) | 18.50 (16.00, 23.50) | 19.00 (14.50, 23.50) | 0.226 |
| Pathological type  Well differentiated  Moderately differentiated  Poorly differentiated | 20 (12.50)  51 (31.88)  89 (55.63) | 4 (7.02)  14 (24.56)  39 (68.42) | 6 (12.00)  16 (32.00)  28 (56.00) | 10 (18.87)  21 (39.62)  22 (41.51) | 0.071 |
| Lymhovascular invasion  negative  positive | 146 (91.25)  14 (8.75) | 47 (82.46)  10 (17.54) | 47 (94.00)  3 (6.00) | 52 (98.11)  1 (1.89) | 0.010* |
| WHO performance status  0  1 | 111 (69.38)  49 (30.63) | 37 (64.91)  20 (35.09) | 37 (74.00)  13 (26.00) | 37 (69.81)  16 (30.19) | 0.594 |
| Lauren type  Intestinal type  gastric type | 66 (41.25)  94 (58.75) | 19 (33.33)  38 (66.67) | 24 (48.00)  26 (52.00) | 23 (43.40)  30 (56.60) | 0.284 |
| Intraoperative blood transfusion  negative  positive | 103 (64.38)  57 (35.63) | 25 (43.86)  32 (56.14) | 36 (72.00)  14 (28.00) | 42 (79.25)  11 (20.75) | ＜0.001* |
| Invasive pattern  INFa  INFb  INFc | 12(7.50)  35(21.88)  113(70.63) | 4(7.02)  14(24.56)  39(68.42) | 5(10.00)  6(12.00)  39(78.00) | 3(5.66)  15(28.30)  35(66.04) | 0.288 |
| K-M grade  low  high | 136(85.00)  24(15.00) | 45(78.95)  12(21.05) | 46(92.00)  4(8.00) | 45(84.91)  8(15.09) | 0.169 |
| Tumour stroma percentage  low  high | 145(90.63)  15(9.38) | 54(94.74)  3(5.26) | 43(86.00)  7(14.00) | 48(90.57)  5(9.43) | 0.296 |
| TNM stage  I  II  III | 48 (30.00)  48 (30.00)  64 (40.00) | 22 (38.60)  14 (24.56)  21 (36.84) | 10 (20.00)  19 (38.00)  21 (42.00) | 16 (30.19)  15 (28.30)  22 (41.51) | 0.290 |
| Dissection  D1  D2  D3 | 118 (73.75)  4 (2.50)  38 (23.75) | 35 (61.40)  3 (5.26)  19 (33.33) | 38 (76.00)  1 (2.00)  11 (22.00) | 45 (84.91)  0 (0.00)  8 (15.09) | 0.057 |
| Recurrence  negative  positive | 90 (56.25)  70 (43.76) | 11 (19.30)  46 (80.70) | 29 (58.00)  21 (42.00) | 50 (94.34)  3 (5.66) | ＜0.001* |
| Mortality  negative  positive | 86 (53.75)  74 (46.25) | 8 (14.04)  49 (85.96) | 29 (58.00)  21 (42.00) | 49 (92.45)  4 (7.55) | ＜0.001* |

K-M grade, Klintrup–Makinen grade; *, indicates that difference is significant (*P*<0.05).

Table 2 Univariate and multivariate Cox proportional hazards analyses of recurrence-free survival an overall survival.

| Variables | Categories | Recurrence-free survival | | | | | | Overall survival | | | | | |
| --- | --- | --- | --- | --- | --- | --- | --- | --- | --- | --- | --- | --- | --- |
|  |  | Univariate analysis | | | Multivariate analysis | | | Univariate analysis | | | Multivariate analysis | | |
|  |  | Risk ratio | 95% CI | P-value | Risk ratio | 95% CI | P-value | Risk ratio | 95% CI | p-value | Risk ratio | 95% CI | p-value |
| Sex | male | Reference group |  |  |  |  |  |  |  |  |  |  |  |
|  | female | 0.575 | 0.340-0.975 | 0.040* | 0.786 | 0.453-1.363 | 0.391 | 0.601 | 0.363-0.998 | 0.049* | 0.791 | 0.467-1.342 | 0.385 |
| Age | ≤60 | Reference group |  |  |  |  |  |  |  |  |  |  |  |
|  | ＞60 | 0.967 | 0.605-1.545 | 0.888 |  |  |  | 1.018 | 0.645-1.606 | 0.940 |  |  |  |
| Size | ＜3 | Reference group |  |  |  |  |  |  |  |  |  |  |  |
|  | 3-5 | 1.249 | 0.703-2.219 | 0.447 |  |  |  | 1.280 | 0.732-2.237 | 0.387 |  |  |  |
|  | ＞5 | 1.170 | 0.614-2.229 | 0.634 |  |  |  | 1.203 | 0.642-2.254 | 0.564 |  |  |  |
| Family | negative | Reference group |  |  |  |  |  |  |  |  |  |  |  |
|  | positive | 1.308 | 0.626-2.732 | 0.475 |  |  |  | 1.247 | 0.598-2.597 | 0.556 |  |  |  |
| Lose weight | negative | Reference group |  |  |  |  |  |  |  |  |  |  |  |
|  | positive | 2.368 | 1.436-3.905 | 0.001* | 1.623 | 0.967-2.726 | 0.067 | 2.423 | 1.487-3.949 | ＜0.001* | 1.562 | 0.933-2.613 | 0.090 |
| location | cardia | Reference group |  |  |  |  |  |  |  |  |  |  |  |
|  | gastric body | 2.103 | 0.490-9.035 | 0.317 |  |  |  | 2.136 | 0.499-9.144 | 0.306 |  |  |  |
|  | [antrum](javascript:void(0);) | 1.240 | 0.300-5.123 | 0.767 |  |  |  | 1.268 | 0.308-5.231 | 0.742 |  |  |  |
|  | total stomach | 4.686 | 0.970-22.649 | 0.055 |  |  |  | 3.933 | 0.815-18.987 | 0.088 |  |  |  |
| Pathological type | well differentiated | Reference group |  |  |  |  |  |  |  |  |  |  |  |
|  | moderately differentiated | 1.659 | 0.622-4.421 | 0.312 |  |  |  | 2.287 | 0.788-6.637 | 0.128 |  |  |  |
|  | poorly differentiated | 2.712 | 1.076-6.836 | 0.034* |  |  |  | 3.624 | 1.306-10.054 | 0.013* |  |  |  |
| Lymhovascular invasion | negative | Reference group |  |  |  |  |  |  |  |  |  |  |  |
|  | positive | 3.521 | 1.831-6.771 | ＜0.001* | 2.015 | 1.025-3.960 | 0.042* | 3.144 | 1.643-6.014 | 0.001* | 1.736 | 0.891-3.382 | 0.105 |
| WHO performance status | negative | Reference group |  |  |  |  |  |  |  |  |  |  |  |
|  | positive | 1.642 | 1.014-2.658 | 0.044* | 1.540 | 0.938-2.529 | 0.088 | 1.672 | 1.048-2.668 | 0.031* | 1.565 | 0.964-2.541 | 0.070 |
| Lauren type | Intestinal type | Reference group |  |  |  |  |  |  |  |  |  |  |  |
|  | gastric type | 1.091 | 0.678-1.756 | 0.719 |  |  |  | 1.200 | 0.752-1.914 | 0.444 |  |  |  |
| Invasive pattern | INFa | Reference group |  |  |  |  |  |  |  |  |  |  |  |
|  | INFb | 0.506 | 0.212-1.206 | 0.124 |  |  |  | 0.446 | 0.193-1.034 | 0.060 |  |  |  |
|  | INFc | 0.537 | 0.254-1.137 | 0.104 |  |  |  | 0.494 | 0.242-1.006 | 0.052 |  |  |  |
| K-M grade | low | Reference group |  |  |  |  |  |  |  |  |  |  |  |
|  | high | 1.406 | 0.770-2.569 | 0.267 |  |  |  | 1.373 | 0.754-2.500 | 0.299 |  |  |  |
| Tumour stroma percentage | low | Reference group |  |  |  |  |  |  |  |  |  |  |  |
|  | high | 0.762 | 0.330-1.760 | 0.525 |  |  |  | 0.860 | 0.395-1.874 | 0.705 |  |  |  |
| TNM stage | I | Reference group |  |  |  |  |  |  |  |  |  |  |  |
|  | II | 0.884 | 0.490-1.597 | 0.684 |  |  |  | 0.803 | 0.450-1.433 | 0.458 |  |  |  |
|  | III | 0.797 | 0.452-1.407 | 0.434 |  |  |  | 0.794 | 0.460-1.371 | 0.408 |  |  |  |
| Dissection | D1 | Reference group |  |  |  |  |  |  |  |  |  |  |  |
|  | D2 | 1.915 | 0.462-7.936 | 0.370 |  |  |  | 2.027 | 0.488-8.413 | 0.331 |  |  |  |
|  | D3 | 2.373 | 1.441-3.909 | 0.001* |  |  |  | 2.286 | 1.405-3.719 | 0.001* |  |  |  |
| EFTS | low | Reference group |  |  |  |  |  |  |  |  |  |  |  |
|  | - moderate | 0.210 | 0.123-0.358 | ＜0.001* | 0.229 | 0.132-0.396 | 0.000* | 0.184 | 0.108-0.312 | ＜0.001* | 0.201 | 0.117-0.346 | ＜0.001* |
|  | high | 0.023 | 0.007-0.076 | ＜0.001* | 0.025 | 0.008-0.083 | 0.000* | 0.026 | 0.009-0.074 | ＜0.001* | 0.028 | 0.010-0.080 | ＜0.001* |

*, indicates that difference is significant (*P*<0.05); CI, confidence intervals; EFTS, elastic fibers in tumor stroma; K-M grade, Klintrup–Makinen grade; Sex, lose weight, lymhovascular invasion, WHO performance status and EFTS were the adjustment factors for multivariate Cox proportional hazards analyses of recurrence-free survival and overall survival, respectively.
